# Prognostic factors and treatment outcomes in pediatric autoimmune encephalitis: a multicenter study

**DOI:** 10.3389/fneur.2024.1441033

**Published:** 2024-09-02

**Authors:** Ahlam Ahmed Abu Melha, Amjad Saad Aldress, Fahad Alamri, Lama Saleh Aljomah, Raid Hommady, Ahmed Al-Rumayyan, Fahad Albassam

**Affiliations:** ^1^Department of Pediatric Neurology, King Abdullah International Medical Research Center, Riyadh, Saudi Arabia; ^2^King Saud Bin Abdulaziz University for Health Sciences, Riyadh, Saudi Arabia; ^3^College of Medicine, King Saud bin Abdulaziz University for Health Sciences, Riyadh, Saudi Arabia; ^4^King Abdullah International Medical Research Center, Riyadh, Saudi Arabia; ^5^Department of Pediatric Neurology, King Fahad Medical City, Riyadh, Saudi Arabia

**Keywords:** autoimmune encephalitis, paraneoplastic syndrome, synaptic receptors, onconeural antibodies, NMDAR antibodies

## Abstract

**Introduction:**

The last few decades have increased our understanding of autoimmune encephalitis (AE). In both the pediatric and adult populations, it proves to be a disease of dramatic acute onset of heterogeneous clinical manifestations, notably encephalopathy with neuropsychiatric symptoms, seizures, and extrapyramidal symptoms. More often, it is triggered by a viral infection in the pediatric age groups, as suggested by the preceding febrile symptoms in over half of cases, and more ostensibly, NMDAR encephalitis post herpes encephalitis. An underlying neoplasm may be present in certain types (i.e., NMDAR encephalitis). The rising rate of antibody detection and subsequent confirmation has been boosted by improved live cellular assay detection methods. The corresponding clinical phenotypes, common underlying malignancies, and histopathological findings have helped improve our management regarding intervention and choice of immunotherapy. New assessment tools such as the Clinical Assessment Scale in Autoimmune Encephalitis (CASE score) have helped improve the objective assessment of impact on cognitive functions (1). Early intervention with immunotherapy (and tumor removal in proven underlying neoplasms) has improved overall outcomes in most presenting patients. But nearly 40% of cases fail to respond to the first tier of treatment (2). The complex interplay between pathogenic autoantibodies, T-cells, B-cells, and cytokines has led to the emergence of additional immunotherapy agents (i.e., tocilizumab and bortezomib).

**Methods:**

In this retrospective observational study of pediatric AE conducted at two tertiary care centers, we observed the clinical characteristics, autoantibody yield, treatment modalities used, and disability scores during presentation and follow-up. Our secondary aim was to delineate prognostic factors for poor outcomes.

**Results:**

Neuropsychiatric symptoms, encephalopathy, and seizures were the predominant manifestations in most of our patients. Younger age groups, refractory seizures, profound encephalopathy, and refractory disease harbored higher disability scores. The group that received combined immunotherapy has shown mitigation of disability score from severe to mild during long-term follow-up, signifying the role of multifaceted immunotherapy in pediatric refractory AE.

**Conclusion:**

Early implementation of combined immunotherapy in refractory cases significantly improved longterm disability scores, in spite of lingering residual effects on neurologic functions, notably cognition, behavior, and speech.

## Introduction

Autoimmune encephalitis (AE) represents a rare yet increasingly recognized disorder affecting both adults and children. It was first reported in 1968 as a paraneoplastic autoimmune encephalitis associated with small-cell lung cancer (1). The incidence of AE has arisen during the past two decades from 0.4 to 1.2/100,000 person-years (2). Its prevalence (13.7/100,000) exceeds that of infectious encephalitis (11.6/100,000) (2). This is partly attributable to the rising number of recognized auto-antibodies and the concomitant underlying inflammatory mediators (3). According to a UK multicenter study, 4% of encephalitis patients exhibited NMDA receptor antibodies, making this illness the second most common cause of immune-mediated encephalitis, second only to acute disseminated encephalomyelitis (4). According to the California Encephalitis Project, anti-NMDA receptor encephalitis was more common than viral encephalitis (2).

The pathogenesis of AE is mainly mediated by antibodies (Abs) to the central nervous system (CNS). AE is broadly categorized based on the location of the targeted antigens. Antibodies targeting intracellular proteins (e.g., Anti-Hu, Ma, Glutamic acid decarboxylase, Amphiphysin, and Ri) are associated with onconeural syndromes (e.g., small-cell lung carcinoma) and are often referred to as onconeural antibodies (3, 5, 6). Antibodies targeting neuronal cell-surface receptors are referred to as synaptic antibodies. These target receptors are involved in synaptic transmission and neuronal signaling (7). These include Anti-N-methyl-D-aspartate receptor (NMDAR) antibodies, leucine-rich glioma inactivated-1 (LGI1), contactin-associated protein-like 2 (Caspr2), alpha-amino-3-hydroxy-5-methyl-4-isoxazolepropionic acid receptor (AMPAR), gamma-aminobutyric acid (GABA)-A and -B receptors, dipeptidyl-peptidase-like protein-6 (DPPX), and glycine receptor (GlyR). The literature has shown the diverse clinical phenotypes characterizing each autoantibody (8).

The diversity in the AE phenotypes and corresponding treatment response has helped impart a better understanding of the underlying neuroinflammatory mechanisms. Earlier implementation of first and second-line immunotherapy had shown better recovery (9). The initial severity of the encephalitis at the time of presentation is a significant prognostic factor. Children presenting with profound encephalopathy, status epilepticus, or coma may face a longer and more complicated recovery process (10). While many children with AE experience significant neurological recovery, some may face persistent cognitive challenges, including difficulties with memory, attention, and executive function (11). These long-term effects highlight the importance of comprehensive neuropsychological assessments and ongoing support. These studies have utilized standardized assessment scales like the modified Rankin Scale (mRS) and Clinical Assessment Scale in Autoimmune Encephalitis (CASE) scores to evaluate neurological function and predict outcomes (12). We are gaining comprehensive insights into prognostic factors and treatment outcomes concerning seronegative and refractory AE. Several multicenter studies, such as those conducted by Qiao et al. (13) and Pruetarat et al. (11), have shed light on prognostic indicators and treatment responses in AE, primarily focusing on adult populations or specific antibody subtypes, such as anti-N-methyl-D-aspartate receptor (NMDAR) encephalitis (11, 13). In NMDAR encephalitis, pediatric AE can be associated with underlying tumors, such as teratomas. The presence or absence of such tumors and their successful treatment can impact long-term outcomes (14, 15). Moreover, investigations into pediatric AE, such as the study in Korea by Shim et al. (16) have demonstrated varying degrees of disease severity and recovery trajectories, emphasizing the need for tailored prognostic assessments in this population.

Despite these advancements, consensus regarding criteria for prognostic factors and treatment outcomes in the pediatric population is further wanting. Given the potential influence of demographic, clinical, and management variables on disease trajectory, a compelling rationale exists to conduct a comprehensive evaluation within our population. Identifying factors influencing treatment response and long-term outcomes is essential for optimizing therapeutic approaches in pediatric AE. By assessing the clinical response to treatment and identifying potential prognostic factors, we aim to enhance our understanding of pediatric AE, facilitating more personalized management strategies and improving long-term outcomes for affected patients. Given the complexities inherent in pediatric AE, there is an urgent need for comprehensive multicenter studies to elucidate prognostic factors and treatment outcomes in this population. This study seeks to address this gap by delineating pediatric AE’s clinical course and prognostic determinants within our institutions, contributing valuable insights to the global body of knowledge on autoimmune encephalitis.

## Materials and methods

### Study setting and duration of study

An observational retrospective cohort study was conducted in two tertiary centers, namely King Abdullah Specialist Hospital and King Fahad Medical City, located in Riyadh, Saudi Arabia.

### Study subjects/participants

#### Inclusion criteria

The study included all patients who were admitted into the pediatric department (in both medical centers, the stipulated age is below14 years), who presented sub-acutely (within days to less than 6 weeks) (3), and whose constellation of symptoms fulfilled the clinical consensus criteria for pediatric autoimmune encephalitis (4).

#### Exclusion criteria

Patients whose clinical and imaging fulfilled criteria for CNS acute demyelinating syndromes (e.g., ADEM, NMO, MS). Patients with rheumatological conditions (Systemic/CNS vasculitis, Behçet’s disease, CNS lupus, Neurosarcoidosis), were excluded. A neuroradiologist in each center reviewed the images for discerning the findings along with reliance of complementary laboratory testing.

#### Recruitment

Patients meeting the inclusion criteria were identified through a comprehensive review of electronic medical records at the participating hospitals. Patients admitted during the period of April 2015 to December 2022 were included. Assuming a conservative estimated proportion (p) of pediatric patients with autoimmune encephalitis based on previous literature or clinical experience (e.g., 50%) and a desired margin of error (d) of 10%, a minimum sample size of 40 pediatric patients was deemed appropriate for this study. This determination considers practical constraints and feasibility, ensuring adequate statistical power while accommodating potential limitations in data availability or patient recruitment.

#### Data collection procedures and tool

Data were collected through meticulous chart review of electronic medical records, employing a self-developed data collection sheet. The collected information encompassed demographic details (age, gender, area of origin), clinical characteristics (i.e., encephalopathy, seizures, speech regression extrapyramidal symptoms etc.), neuroimaging findings, serologic and CSF tests. All study subjects had undergone serologic and CSF autoimmune encephalitis panel testing which was conducted at Bioscientia Laboratory (a send-out facility). The method used for antibody testing was indirect immunofluorescence testing. Study subjects were also tested for Myelin Oligodendrocyte Glycoprotein (MOG) IgG antibodies and Aquaporin-4 IgG antibodies. Rheumatological and vasculitis markers in the serum were included. Neuroimaging consisted of neuroinflammatory protocol MRI brain (which included diffusion weighted, T2/FLAIR, and contrast guided T1 images). Management strategies (first-line, second-line, maintenance therapy), and long-term follow-up outcomes (for relapses and CASE scores). To ensure reliability in data collection, inter-rater reliability tests were conducted among research team members. This involved independent chart reviews by multiple team members followed by comparison and assessment of agreement on extracted data. Any discrepancies or disagreements were resolved through discussion and consensus among team members. Additionally, periodic quality checks were conducted throughout the data collection process to maintain consistency and accuracy in data extraction. These measures aimed to enhance the reliability of collected data and minimize potential biases in the study findings. The clinical outcomes of patients with autoimmune encephalitis were assessed according to their Clinical Assessment Scale in Autoimmune Encephalitis (CASE) scores categorized as excellent (0–4), moderate (5–9), or poor (10–27). CASE scores quantitatively measure disease severity and clinical outcomes in autoimmune encephalitis. It includes nine items: seizures, memory dysfunction, psychiatric symptoms, consciousness, language problems, dyskinesia/dystonia, gait instability, and ataxia. It has subitem scores from 0 to 3 for each key item, with a sum of nine keys and a sum score of 27. Higher CASE scores indicate greater symptom burden and functional impairment (17, 18).

#### Ethical approval

The study’s ethical approval was obtained from the institutional review board of the principal investigator’s institution. Given the study’s retrospective nature, written consent was waived, ensuring stringent adherence to patient privacy and confidentiality protocols.

#### Data analysis

SPSS version 25 (IBM Corp, Armonk, New York) was used to analyze the data. Descriptive statistics, including mean and standard deviation (SD) for continuous variables, and frequencies and percentages for categorical variables, were calculated to summarize the characteristics of the study population. Statistical tests such as chi-square or Fisher’s exact test were employed to analyze categorical variables, ensuring robust statistical inference. A significance level of *p* < 0.05 was used to determine statistical significance.

## Results

**Figure 1:**
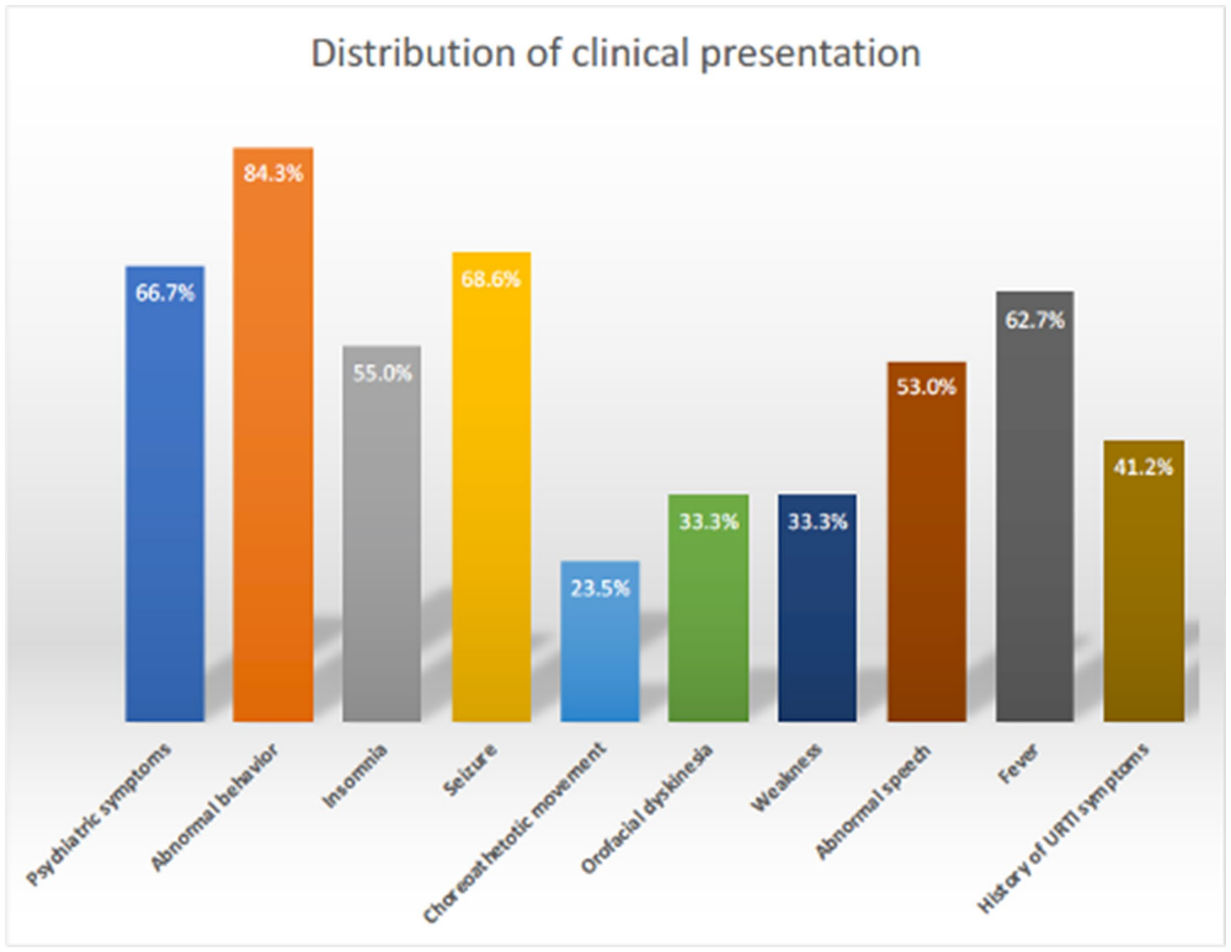
Clinical presentation distribution of AE in children.

The sociodemographic and clinical characteristics of the 51 patients are depicted in Table 1. The cohort’s mean age was 9.8 years, with a slight male predominance (53%) over females (47%). Most patients (70.5%) did not have comorbidities. The majority hailed from the central region of Saudi Arabia (53%). Psychiatric symptoms (i.e., delusions, hallucinations, disorganized thinking) (66.7%) and abnormal behavior (84.3%) were commonly observed, indicating the predominance of neuropsychiatric manifestations, followed by encephalopathy (78.4%), seizures (68.6%), speech regression (58.8%), and insomnia (55%) (Figure 1). Orofacial dyskinesia was the most common extrapyramidal feature observed (23.5%), followed by choreoathetosis (23.5%).

**Table 1:** Sociodemographic and clinical characteristics.

Sociodemographic details		*N* (%)
Age (Mean ± SD)	9.8 ± 4.1 years	
Gender	Male	27 (53)
	Female	24 (47)
Comorbidities	None	36 (70.5)
	Yes	15 (29.5)
City of origin in KSA	Central	27 (53)
	North	3 (5.8)
	South	7 (13.7)
	West	5 (9.8)
	Non- Saudi	9 (17.6)
Presenting symptoms		
Psychiatric symptoms	Yes	34 (66.7)
	No	17 (33.3)
Abnormal behavior	Yes	43 (84.3)
	No	8 (15.7)
Insomnia	Yes	28 (55)
	No	23 (45)
Seizure	Yes	35 (68.6)
	No	16 (31.4)
Choreoathetosis	Yes	12 (23.5)
	No	39 (76.5)
Orofacial dyskinesia	Yes	17 (33.3)
	No	34 (66.7)
Weakness	Yes	17 (33.3)
	No	34 (66.7)
Abnormal speech	Yes	27 (53)
	No	24 (47)
Fever	Yes	32 (62.7)
	No	19 (37.3)
History of URTI symptoms	Yes	21 (41.2)
	No	28 (55)
	COVID-19	2 (3.9)
Associated symptoms clinically		
Encephalopathy	Yes	40 (78.4)
	No	11 (21.6)
Weakness	Yes	12 (23.5)
	No	39 (76.5)
Orofacial dyskinesia	Yes	12 (23.5)
	No	39 (76.5)
Choreoathetosis	Yes	8 (15.7)
	No	43 (84.3)
Incomprehensive speech	Yes	30 (58.8)
	No	21 (41.2)
Other finding	Yes	26 (51)
	NO	25 (49)
PICU admission	Yes	23 (45)
	No	28 (55)
CBC findings (Mean ± SD)	WBC	9.1 ± 4.3
	Hgb	46.5 ± 39.3
	Platelets	359.7 ± 132.3
Inflammatory markers Median (IQR)	ESR	20 (6–39)
	CRP	1.8 (0.46–8)
VW factor	Normal (50–200)	18 (35.3)
	High >200	3 (5.9)
	Not done	26 (51)
	None	4 (7.8)
CSF result Median (IQR)	WBC	1 (0–3)
	Protein	0.22 (0.18–0.28)
CSF PCR and culture	Negative	48 (94)
	Positive HSV	2 (3.9)
	Positive HHV6	1 (1.9)
CSF oligoclonal bands	Negative	27 (53)
	POSITIVE	5 (9.8)
	Not Done	19 (37.3)
CSF antibodies	Negative	29 (56.8)
	Not done	4 (7.8)
	anti-NMDA receptor Ab positive	17 (33.3)
	GAD65 AB: High (241.3)	1 (1.9)
Serum antibodies	Negative	26 (51)
	Positive	23 (45)
	Not done	2 (4)
Brain MRI findings	Normal	17 (32)
	Subcortical T2/FLAIR hyperintensities	23 (43.4)
	Atrophy	3 (5.6)
	Cortical T2 hyperintensities	5 (9.4)
	Combined cortical and white matter T2 hyperintensities	3 (5.6)
	Cortical malformation/Gray and White matter changes	1 (1.8)
Initial EEG findings	Normal	12 (19.3)
	Unremarkable	1 (1.6)
	Epileptic	18 (29)
	Background non-epileptiform abnormalities	29 (46.8)
	Electrographic seizures	2 (3.2)
Pulse steroids	Yes	50 (98.1)
	No	1 (1.9)
Oral steroids	Yes	35 (68.6)
	No	16 (31.4)
IVIG	Yes	45 (88)
	No	6 (11.8)
Monthly IVIG	Yes	23 (50.1)
	No	28 (55)
Plasmapheresis	Yes	21 (41.2)

Laboratory findings showed that Inflammatory markers such as ESR and CRP were elevated, indicating an underlying inflammatory process. The median WBC count in cerebrospinal fluid (CSF) was within normal limits, suggesting a lack of overt infection. Most CSF PCR and culture results were negative (94%), indicating a low infectious burden. Of the patients who met the criteria for probable autoimmune encephalitis, 18 (35.2%) patients were found to be positive for autoantibodies, confirming their status as definite autoimmune encephalitis. Of these, 17/18 (94%) were NMDA receptor antibodies, and 1/18 (5%) were GAD65 antibodies in the CSF.

Brain MRI revealed abnormalities in a significant proportion of patients, including white matter changes (43.4%) and cortical malformations (1.8%). Initial EEG findings indicated epileptic activity in a notable percentage of cases (32.3%). Pulse steroids (methylprednisolone 20–30 milligram per kg, or 1 gram per dose intravenous daily for 3–5 days) were commonly administered (98.1%) as first-line treatment. Concomitant oral steroids (prednisolone 1.5–2 milligram per kg, with gradual tapering over 4–6 weeks) were used in (68.6%). Intravenous immunoglobulin (IVIG) therapy (2 gram per kg per course over 2–4 days) were used in (88%) of patients. Therapeutic Plasmapheresis (average 5–7 cycles) was utilized in a considerable proportion of cases (41.2%). A subset of patients received monthly IVIG (1–2 gram/kg) infusions (50.1%), indicating a chronic treatment approach.

**Figure 2:**
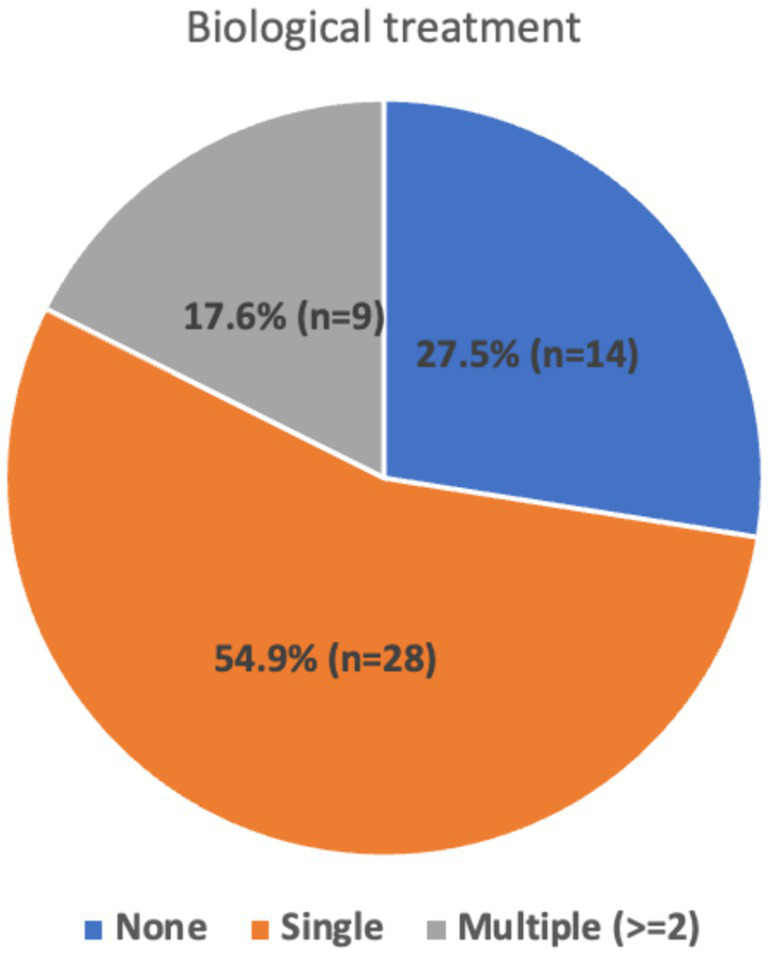
Distribution of biological treatments.

The analysis showed that among the participants, 27.5% received no second-line immunotherapy, 54.9% received a single second-line immunotherapy agent, and 17.6% received 3rdline treatment (Figure 2). The distribution of different immunotherapy agents used is given in Table 2. For second-line immunotherapy, Rituximab (anti-CD20 monoclonal antibody therapy) was the most used agent (56.9%), followed by Tocilizumab (Interleukin-6 antagonist) (9.8%). The remaining agents encompassed Bortezomib (Proteasome inhibitor), intrathecal methotrexate/dexamethasone, and mycophenolate mofetil (2.0% each). Within the 3rd line treatment group, Rituximab and Tocilizumab were the most used (5.9% each), followed by Mycophenolate mofetil (3.9%) and IT Methotrexate (2%). 5.9% continued maintenance immunotherapy, of which 3.9% comprised Rituximab and 2% Azathioprine.

**Table 2:** Distribution of different biological agents used.

Second line immunotherapy	No treatment	14	27.5
	Bortezomib	1	2.0
	IT Methotrexate	1	2.0
	MMF	1	2.0
	Rituximab	29	56.9
	Tocilizumab	5	9.8
Third line immunotherapy	No treatment	42	82.4
	IT Methotrexate	1	2.0
	Mycophenolate Mofetil	2	3.9
	Rituximab	3	5.9
	Tocilizumab	3	5.9
Maintenance immunotherapy	No treatment	48	94.1
	Azathioprine	1	2.0
	Rituximab	2	3.9

The impact of immunotherapy agents on CASE scores at presentation and follow-up was analyzed (Table 3). Results revealed significant discrepancies in scores among the treatment groups both at presentation (*p* = 0.020) and follow-up (*p* = 0.018). At presentation, individuals receiving multiple immunotherapies demonstrated markedly higher mean scores (14.89 ± 7.149) compared to those on a single treatment (9.39 ± 6.471) and those without any treatment (7.43 ± 4.450). Similarly, at follow-up, patients undergoing multiple biological treatments exhibited notably elevated mean scores (8.00 ± 7.018) compared to those on a single treatment (3.82 ± 4.489) and those without any treatment (2.36 ± 2.023).

**Table 3:** Comparison of CASE scores based on biological treatment.

Situation	Treatment		CASE scores		*p*-value
		*N*	Mean	Std. deviation	
At presentation	No Rx	14	7.43	4.450	0.020
	Single	28	9.39	6.471	
	Multiple	9	14.89	7.149	
At follow-up	No Rx	14	2.36	2.023	0.018
	Single	28	3.82	4.489	
	Multiple	9	8.00	7.018	

A *p*-value <0.05 is considered statistically significant.

**Figure 3:**
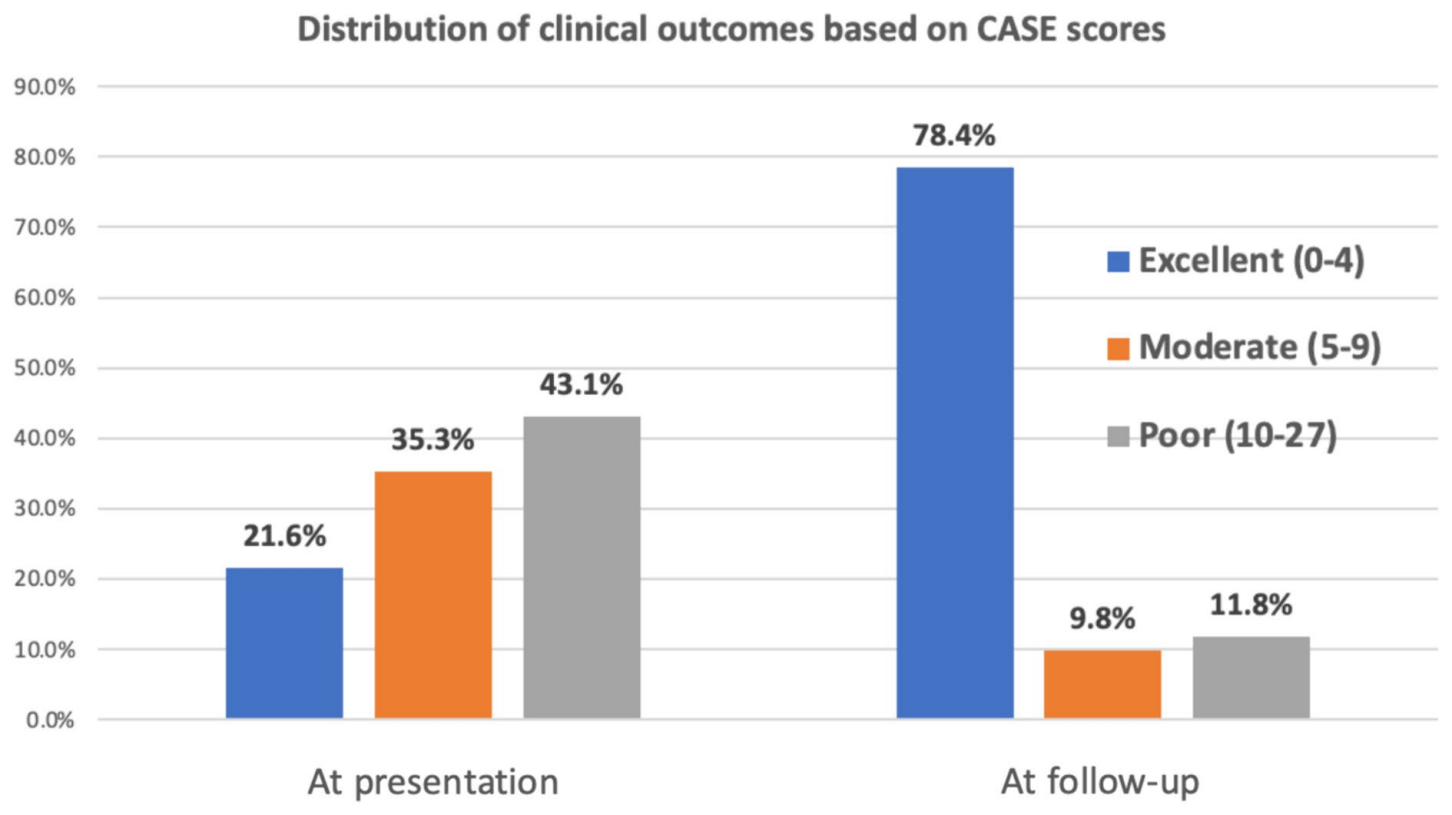
Distribution of clinical outcomes.

The analysis revealed a significant improvement in patient outcomes over time. At presentation, a substantial proportion of patients fell into the poor category (43.1%), followed by moderate (35.3%) and excellent (21.6%) categories. During follow-up, however, there was a remarkable shift toward better outcomes, with most patients (78.4%) achieving an excellent CASE score. Conversely, only a small percentage remained in the moderate (9.8%) and poor (11.8%) categories (Figure 3).

## Discussion

The clinical presentations of AE differ in children from adults because of the evolution of neural circuits, neuroreceptor density, and myelination in the former (10). Children with AE often exhibit multifocal neuropsychiatric symptoms rather than isolated symptoms and are susceptible to long-term mild disabilities (e.g., learning difficulties). The relative prevalence of certain illnesses underlines the significance of immune-mediated encephalitis. The current study’s findings showed that encephalopathy was prevalent in 78.4% of our patients and psychiatric symptoms in 66.7%. Neuropsychiatric symptoms typically manifest suddenly in children who were previously healthy and had AE (19, 20). Distinguishing primary psychiatric disorders from AE-related psychiatric manifestations can be challenging, especially in the absence of overt neurological symptoms. Psychiatric symptoms encompass a broad spectrum of manifestations, including mood disturbances, psychosis, anxiety, and behavioral changes. However, these symptoms may be underreported or misinterpreted, particularly in young children or patients with communication difficulties, and misdiagnosis or delayed diagnosis may occur, leading to inappropriate treatment or prolonged morbidity. New onset psychosis in a previously healthy child should prompt the necessary work-up to investigate underlying immunological, metabolic, and infectious causes. The past few years have shown growing evidence of acute onset psychosis in children that is immune-mediated, particularly with N-Methyl-D-Aspartate (NMDA) receptor, Leucine-rich glioma inactivated-1 (LGI-1), contactin-associated protein-like 2 (CASPR2), and Glutamic Acid Decarboxylase (GAD65) (21, 22). The presence of focal and generalized seizures was another predominating feature in our patients. Epilepsy management in AE can be challenging, as they may demonstrate pharmacoresistance (23–26). Increasing evidence suggests a link between inflammation and drug-resistant epilepsy. The latter has been observed in AE, FIRES, NORSE, Rasmussen Encephalitis, and Focal Cortical Dysplasia. which elaborates the role of inflammation in epilepsy, with their respective different underlying pathogenesis and prevailing immune mediators (27). Understanding the underlying pathogenesis influences treatment decisions. For cases such as febrile-infection-related-epilepsy (FIRES), new-onset refractory status epilepticus (NORSE), and super refractory status epilepticus (SRSE), an underlying cytokine storm plays a pivotal role. Anakinra (IL-1 antagonist) has been found to reduce seizure burden in those cases effectively (28). Tocilizumab (IL-6 antagonist) use has been limited to NORSE and SRSE cases with similar effective results in seizure control. However, there was a 20% risk of opportunistic infections. Other entities responsible for immunomodulation in Refractory status epilepticus include the Ketogenic diet and cannabinoids. Alpha-4-beta-1 antagonist (natalizumab) and tumor necrosis factor inhibitors (etanercept) have been found to reduce seizure frequency in Rasmussen encephalitis (28). In AE, where humoral immunity is prominently involved, rituximab (anti-CD20) is commonly used as a second-line treatment due to its relative tolerance and effectiveness. One has also to consider other possible etiologies for refractory seizures and poor response to treatment, such as structural, toxic, metabolic, and genetic etiologies.

In most cases, symptoms will last for some time after the start of treatment (29, 30). AE differs from pediatric acute-onset neuropsychiatric syndrome (PANS) in that PANS patients frequently go through a cycle of symptoms that worsen and improve quickly, often within hours or days, even without treatment. For the PANDAS/PANS group entities, several treatment options have been used, including adenotonsillectomy, antibiotics (penicillin V, Azithromycin), anti-inflammatory drugs (i.e., cyclooxygenase inhibitors, corticosteroids, IVIG, plasma exchange) (31). Evidence indicates that more than one-third of patients with AE exhibit abnormal movements, encompassing a spectrum of manifestations such as ataxia, chorea, dystonia, myoclonus, or tremor (20, 32–34). Additionally, cognitive impairment emerges as a predominant and defining symptom in most AE cases. Consequently, diagnosing AE becomes questionable in individuals who demonstrate confirmed intact cognitive abilities, which serves to differentiate AE from PANS, where cognition typically remains unaffected. Assessing memory impairments in young children poses inherent challenges; however, developmental regression, language regression, or speech difficulties can be discernible indicators of pediatric AE (21). These clinical nuances underscore the intricate diagnostic considerations and the importance of a comprehensive evaluation encompassing cognitive and behavioral domains in suspected cases of AE.

Changes in behavior, including irritability, hyperactivity, hypersexuality, sleeplessness, and outbursts of rage, are prevalent in children with AE (29, 35). Our findings showed that 84.3% of our patients had some aspect of abnormal behavior. As reported by previous studies, psychiatric symptoms in AE exhibit a broad spectrum, ranging from mood swings and mild personality changes to severe psychosis, affecting over 50% of AE patients (15, 17, 36). However, the occurrence of new-onset psychosis in children below the age of 13 is infrequent and raises concerns regarding an underlying medical etiology rather than a primary psychiatric disorder (24, 26, 34). Hence, diligent assessment for cognitive impairments, seizures, movement abnormalities, or other neurological manifestations becomes paramount when evaluating children presenting with acute psychiatric symptoms, as these features strongly indicate a potential diagnosis of AE.

In our findings, patients receiving multiple immunotherapies had substantially higher mean CASE scores than those receiving a single second-line agent and those did not require any. This suggests that patients with more severe disease manifestations or refractory symptoms (failure to respond to first and second-line immunotherapy) are more likely to receive multiple immunotherapy agents. Yang et al., in their review of the 3rd-line treatment approach of refractory autoimmune encephalitis, have underlined the multiple underlying pathogenic mechanisms and the growing treatment repertoire for tackling the various immune mechanisms of autoimmune encephalitis with its diverse manifestations. No clear consensus exists emphasizing long-term benefits versus treatment risks (37). Unresponsive or Refractory autoimmune encephalitis cases may require long-term immunosuppression with the immunotherapy of choice for long-term stability and improvement. The results of our study coincide with the existing literature that refractory autoimmune encephalitis entails a worse disability outcome. It further adds that combined immunotherapy for refractory cases helps improve overall disability from severe to milder disability outcomes. This knowledge advocates further studies toward refining the management consensus for AE.

### Limitations

The study possesses several potential limitations that warrant consideration and cautious interpretation of the findings. The study’s retrospective nature introduces inherent biases and limitations, such as incomplete data collection, reliance on medical records, and the inability to control variables that may influence outcomes. The lack of a control group hinders the ability to compare outcomes between treated and untreated patients or to evaluate the effectiveness of different treatment modalities. Without a control group, it is challenging to determine whether observed outcomes are attributable to the treatment or other factors. Multiple confounding variables may influence treatment outcomes, including patient demographics, disease severity, comorbidities, and concurrent therapies. Without controlling for these factors, it is difficult to ascertain the intervention’s true effect on the outcomes of interest. The study may be subject to selection bias, as patients included in the analysis may not represent the broader population with autoimmune encephalitis. Patients with more severe or refractory disease may be overrepresented, skewing the results toward poorer outcomes. The study’s small sample size limits the statistical power and generalizability of the findings. Small sample sizes increase the risk of false negatives and limit the ability to detect significant associations or differences between groups. The generalizability of the study results is, therefore, limited, as the findings may not be applicable to a wider population beyond the specific sample analyzed.

## Conclusion

In conclusion, our study provides a comprehensive overview of pediatric autoimmune encephalitis, revealing a high prevalence of neuropsychiatric manifestations, underscoring its profound impact on the central nervous system. Additionally, prevalent symptoms such as insomnia, seizures, encephalopathy, and movement disorders were noted. Laboratory findings supported the autoimmune etiology, with elevated inflammatory markers and positive CSF antibodies. Brain imaging and EEG findings were consistent with AE pathology. Analysis of CASE scores indicated significant differences among treatment groups, with higher scores observed in patients receiving multiple biological treatments. Despite this, there was a notable improvement in outcomes over time, with most patients achieving excellent CASE scores at follow-up. Further research is necessary to understand underlying mechanisms better and optimize treatment strategies refractory AE cases.

## Data Availability

The raw data supporting the conclusions of this article will be made available by the authors, without undue reservation.
